# Anisakidae parasites in frozen fish fillets intended for human consumption

**DOI:** 10.7705/biomedica.6533

**Published:** 2022-12-01

**Authors:** Patricia Betancourth, Jairo Gómez, Jorge A. Fernández-Silva, Juliana González

**Affiliations:** 1 Grupo Centauro, Escuela de Medicina Veterinaria, Facultad de Ciencias Agrarias, Universidad de Antioquia, Medellín, Colombia Universidad de Antioquia Grupo Centauro, Escuela de Medicina Veterinaria Facultad de Ciencias Agrarias Universidad de Antioquia, Medellín Colombia; 2 Corporación Colombiana de Investigación, AGROSAVIA, Bogotá, D.C., Colombia Corporación Colombiana de Investigación AGROSAVIA Bogotá, D.C. Colombia

**Keywords:** Anisakiasis, parasite, zoonoses, fishes, foodborne diseases, anisakiasis, parásito, zoonosis, peces, enfermedades transmitidas por los alimentos

## Abstract

**Introduction:**

Anisakiasis is a human parasitic disease caused by the consumption of raw fish or shellfish containing larvae of the Anisakidae family. It is currently considered an emerging disease of public health interest.

**Objective:**

To identify the presence of larvae of the Anisakidae family in samples of frozen raw fish fillets intended for human consumption in markets in Medellín and its metropolitan area in Antioquia, Colombia.

**Materials and methods:**

A cross-sectional study was carried out, in which larvae of the Anisakidae family were detected and identified in frozen raw fish fillets from three representative markets in Medellín and its metropolitan area. A total of 384 ready for consumption fillets were analyzed (197 sawfish, 137 salmon, 37 tuna, and 13 hake), using the pressing and ultraviolet light method. Taxonomic keys were used to identify the collected parasites and to establish its genus. Conventional PCR and Sanger sequencing was performed to determine the species.

**Results:**

Four larvae were found in 4 of the 384 (1.04%) fillets (CI_95%_ 1.04 ± 1.01%). The species of fish in which the larvae were found was sawfish (*Scomberomorus* spp.) and the genus and species of the larvae was established as *Anisakis pegreffii*.

**Conclusions:**

According to the study, the presence of *Anisakis* parasites in frozen raw fish fillets in the influence area is evident.

## Resumen

**Introducción.:**

La anisakiasis es una infección producida por parásitos de la familia Anisakidae, transmitida a los humanos por el consumo de pescado o mariscos crudos. En la actualidad, se considera una enfermedad emergente de interés en salud pública.

**Objetivo.:**

Identificar la presencia de larvas de la familia Anisakidae en muestras de filetes de pescado crudo congelado destinados a consumo humano, en mercados de Medellín y su área metropolitana en Antioquia (Colombia).

**Materiales y métodos.:**

Se realizó un estudio transversal, en el cual se buscó la presencia de larvas de la familia Anisakidae en filetes de pescado crudo congelado de tres mercados representativos de Medellín y su área metropolitana. Se analizaron 384 filetes listos para el consumo (197 sierras, 137 salmones, 37 atunes y 13 merluzas). Cada filete fue analizado mediante el método de prensado y luz ultravioleta. Los parásitos recolectados se identificaron a partir de claves taxonómicas para establecer el género, así como PCR convencional y posterior secuenciación Sanger, para determinar la especie.

**Resultados.:**

Se encontraron 4 larvas en 4 de los 384 filetes (1,04 %) (IC_95%_ 1,04 ± 1,01 %). Las larvas encontradas fueron identificadas como *Anisakis pegreffi* y el tipo de pescado en el cual se encontraron fue la sierra (*Scomberomorus* spp.)

**Conclusiones.:**

De acuerdo con el estudio realizado, se evidencia la presencia de parásitos anisákidos en filetes de pescado crudo congelado en el área de influencia.

Anisakidosis is an emerging ichthyozoonotic parasitic disease with a worldwide distribution caused by nematodes of the Anisakidae family. This parasitosis is transmitted by the consumption of raw or undercooked fish preparations ^1^^,^^2^. The main genera and species of the Anisakidae family of public health significance are *Anisakis simplex, Pseudoterranova decipiens*, and *Contracaecum osculatum*^3^. Due to the acute and non-specific symptoms that it produces, such as abdominal pain, vomiting or diarrhea, the disease can be classified as nonspecific given that it can be confused with other diseases ^4^. Depending on the species of Anisakidae worms, the infection may present with particular characteristics.

In the case of *A. simplex*, it is common to find allergic reactions that can manifest with urticaria and respiratory distress ^5^, while gastrointestinal symptoms are mainly observed if the disease is caused by *P. decipiens* and *C. osculatum*^3^^,^^6^.

The consumption of raw fish in different preparations has been considered a cause of parasitosis in several cultures around the world. However, in countries like Colombia, where this type of consumption is not culturally common, the disease is currently increasing, probably due to the gastronomic influence of countries that include raw fish in their preparations. These changes are triggering the presence of infections by *Anisakis* spp. in the country ^7^^,^^8^.

There is one report of anisakiasis in humans in Colombia, as notified by the *Instituto Nacional de Salud* in 2019 ^2^. However, there are different research approaches that have demonstrated the presence of these parasites in this country ^3^. In addition, there is no epidemiological surveillance system directed to the diagnosis and control of anisakiasis ^2^, making it difficult to find and follow the number of cases that may occur ^9^^,^^10^. An early identification of this type of parasitosis is important, since the infection with some species of the *Anisakis* genus can trigger a type I hypersensitivity reaction, anaphylactic shock, and respiratory distress ^11^.

In Colombia, in the Pacific Coast and limits with Ecuador, previous studies have reported the presence of anisakids in many of the species studied using taxonomic and molecular diagnosis, ^12^^,^^13^. Due that the consumption of raw fish dishes is unavoidable, the risk of acquiring the infection increases, considering that the presence of these parasites has been reported in the country. Therefore, its circulation will depend on several factors; the most important are fish conservation at -20 °C for seven days and its preparation (cooked at 63 °C) ^14^. The lack of knowledge of the population about the prevention of the disease, conditions the presentation of cases in Colombia. Therefore, this study aimed to identify the presence of larvae of the Anisakidae family in samples of frozen raw fish fillets intended for human consumption in markets in Medellín and its metropolitan area in Antioquia, Colombia.

## Materials and methods

### Methodological design

In this cross-sectional study, 384 samples of frozen raw fish fillets were obtained from three main markets located in Medellín and its metropolitan area (*Plaza Minorista José María Villa, Central Mayorista de Antioquia, Placita de Flórez*). According to the *Departamento Administrativo Nacional de Estadística* (DANE), these places are the most visited by fish consumers and the main food suppliers in Antioquia, the collected samples are representative of this population ^16^.

The sample size was calculated to estimate a single proportion with an expected proportion in the population of 50% (due to unknown of the frequency of parasites of the Anisakidae worms in frozen fish in the studied area). Confidence intervals of 95% and estimated errors of 5% were also calculated ^15^.

The fish species included in the study were defined according to the proportion of tons landed in Antioquia, according to the *Autoridad Nacional de Acuicultura y Pesca* (AUNAP). In this way, sawfish (*Scomberomorus* spp.) represented 52% (n=197), salmon (*Salmo salar*) by 35% (n=137), tuna (*Thunnus tuna*) by 10% (n=37), and hake (*Merluccius merluccius*) by 3% (n=13). Subsequently, the number of collected samples for each market was also established: 61% (n=235) of the samples should be obtained from the *Plaza Minorista José María Villa*, 26% (n=97) from the *Central Mayorista de Antioquia*, and 13% (n=52) from the *Placita de Flórez*. The total number of frozen fillets by species and store to be sampled was obtained from the information about the species landed and the number of stores within each market. It is worth mentioning that the selection of the stores was done randomly. Once this was defined, the total number of fillets to be purchased in each market was divided in such a way that the same number of fillets was obtained for each one of them.

### Samples

Fillets of approximately 200 g of each type of frozen fish were collected. No gutted fresh fish, dry fish or in any other preparation was included in the study. The fish species were chosen because they are frequently used in raw or partially sealed preparations, and for their marketing potential, according to data reported by the *Sistema del Servicio Estadístico Pesquero* (SEPEC) for the department in 2017.

### Identification Anisakidae worms

After being purchased, the frozen raw fish fillets were transported immediately at 4 °C to the *Laboratorio de Parasitología Especial* of the *Facultad de Ciencias Agrarias*, *Universidad de Antioquia* in Medellín. The samples underwent a process of progressive thawing at room temperature. Subsequently, the fillets were placed in transparent 14 × 20 cm plastic bags and pressed into 1-2 mm thick layers using a hydraulic pressing device. Once the thin layer of the fillet was obtained, it was first inspected with white light and later, under a UV light source (366 nm), according to the recommended method for frozen samples ^17^.

### Identification of Anisakidae larvae genus

Once the larvae were identified, they were extracted with dissection forceps and preserved in 70% ethanol ^18^ for subsequent taxonomic identification, which was carried out using thermoionic scanning electron microscopy (JEOL-JSM 6490LV, JEOL, Japan) in the laboratory of the *Centro de Microscopía Avanzada* in the *Sede de Investigación Universitaria* (SIU) of the *Universidad de Antioquia*, in Medellín (Colombia). The taxonomic identification was carried out considering the taxonomic keys for the genus as previously proposed ^18^^,^^19^.

### Identification of Anisakidae larvae species

To determine the species of the found larvae, DNA extraction was carried out using a combination of an in-house extraction method (Proteinase K and B-mercaptoethanol), and (MoBio LaboratoriesInc., CA, USA). The amount of extracted DNA was quantified using the Nanodrop 2000 Spectrophotometer^™^ (Thermo Fisher Scientific, USA), obtaining concentrations between 244 ng/µl, and quality ratios of 260/280 and 260/230 of 1,8 to 2. The pair of primers 211B (5′-TTTTCTGGTTATATGGATTGATTTCA-3') and 210 (5'-CACCAACTCTTAAAATTATC-3'), expecting an amplified fragment of around 500 bp, and NC2 (5'-TTAGTTTCTTTTCCTCCGCT-3'), expecting an amplicon size of aproximately 900-1000 bp), were used to carry out the endpoint polymerase chain reaction (PCR) Amplified products were visualized in 2% agarose gels.

### Sequencing

Considering the size of the expected DNA fragments, the first PCR product (amplified with the 221B/210 primers) was only sequenced in one direction and the second fragment (targeted with NC5/NC2 primers) was sequenced in both directions. To carry out the Sanger sequencing process, a purification of 5 µl of the amplified PCR products was initially cleaned up with an exonuclease 1 plus alkaline phosphatase (ExoSAP, Thermo Fisher Scientific), which allows the removal of phosphate groups from dNTPs and primers’ single strings. The cyclic sequence reaction (amplification and labeling with fluoro-labeled dNTPs) was performed using the Bigdye Terminator Cycle Sequencing kit^™^ (Applied Biosystems, Thermo Fisher Scientific, Massachusetts, USA), employing the first fluor of each of the primers of the previously amplified regions as templates.

Finally, the sequence purification was carried out with the BigDye XTerminator Purification kit^™^ (Applied Biosystems, Thermo Fisher Scientific, Foster City, USA). The cleaned products were run on the ABI 3500 Genetic Analyzer^™^ (Applied Biosystems, Foster City, USA), through the Data Collection software 3 and visualized on the Sequencing Analysis program, version 5.4 (Applied Biosystems, Thermo Fisher Scientific, USA), which allowed the evaluation of the raw data (raw view), the quality value (assigned base probability), and the resulting electropherogram.

### Exploration of associations between variables

A semi-structured interview was conducted to the fish sellers at each selected store to obtain information about the place of origin, market, as well as type of fish (table 1). The information was documented in a data capture form, and furtherly used to explore associations between the presence of *Anisakis* spp. and the other variables of interest (table 2).

**Table 1:** Analyzed variables obtained from the semi-structured interview

Variable	Description	Categories	Variable type*	Question made to fish sellers during the semi-structured interview
Place of origin	Geographic place from where the analyzed fish fillet came from according to seller information.	National (Buenaventura Bahía Solano, Santa Marta) Imported (Ecuador, Chile)	Qualitative Nominal	What is the origin of the fish you are currently selling?
Market	Place of commercialization in Medellín and the metropolitan area	Plaza Minorista José María Villa Central Mayorista de Antioquia Placita de Flórez	Qualitative Nominal	n. a.
Type of fish	Common and scientific name of fish sampled	Sierra (*Sawfish*) Salmon (*Salmo salar*) Tuna (*Thunnus*) Hake (*Merluccus merlusus*)	Qualitative	Is the fish you currently sell gutted and/or fresh?
			Nominal	

* Nominal scale for all variables

n. a.: not applicable

**Table 2:** Distribution of *Anisakis* spp. in frozen raw fish fillets samples (N=384), collected in three markets in Medellin and its metropolitan area (Antioquia, Colombia).

Variable	Category	Diagnosis of anisakids		Total (%)	Association		CI_95%_
		Pos (%)^5^	Neg (%)		*X* ^2^ **= 0.05**	Fisher´s	
Place of origin	National^1^	4 (1.73)	231 (98.29)	235 (61.19)	0.107		1.73 ± 1.30
	Imported^2^	0	149 (100)	149 (38.81)			
Market	Plaza Minorista	4 (1.73)	230 (98.29)	234 (60.93)	0.109		1.73 ± 1.30
	José María Villa		150 (100)	150 (39.07)			
	Other^3^	0					
Type of fish	Sawfish	4 (2.03)	193 (97.96)	197 (51.30)	0.050	0.068	2.03 ± 1.40	Other^4^	0	187 (100)	187	(48.70)		

^1^ Buenaventura, Bahía Solano, Santa Marta

^2^ Ecuador, Chile

^3^ Central Mayorista de Antioquia, Placita de Flórez

^4^ *Salmo salar* (salmon), *Thunnus tuna* (tuna), *Merluccus merlusus* (hake).

^5^ Positivity to parasites of the genus *Anisakis* spp., according to the visualization technique of larvae by means of the pressing method and ultraviolet light.

The purchase of the fillets was made in such a way that the seller was unaware of the purpose of the study, choosing the product for sale without any influence from the researchers.

### Statistical analysis

The collected data was recorded in an Excel 2010 sheet (Microsoft, Redmond, USA). The study variables were presence of the larvae (presence, absence), place of origin (national, imported), market (*Plaza Minorista José María Villa, Central Mayorista de Antioquia, Placita de Flórez*), and type of fish (sawfish, salmon, tuna, hake). The results were analyzed by means of descriptive statistics, using frequency tables and percentages. Bivariate analyses were performed using the c^2^ test to explore the association between the presence of Anisakidae larvae and the other study variables. This association was confirmed using Fisher’s exact test. In both cases, a p value <0.05 was established as statistically significant. Statistical analyses were performed using Stata 16.0^™^ (StataCorp, College Station, TX, USA) ^20^.

### Ethical aspects

The present study was proposed and carried out under an expedited endorsement from the *Comité Ético de Experimentación Animal* (CEEA), *Universidad de Antioquia* (Minutes N°. 123, April 2^nd^, 2019).

## Results

### Frequency of Anisakidae larvae and possible associations with other study variables

Four out of 384 samples analyzed contained Anisakidae larvae, with a frequency of 1.04% (CI_95%_: 1.04 ± 1.01%) (table 2). Positive samples were identified in sawfish species that came from the port of Buenaventura (Colombia). No statistical association was found between the presence of *Anisakis* spp. and the explored variables (e.g., place of origin, market, type of fish) (table 2).

### Identification of Anisakidae larvae genus

**Figure 1:**
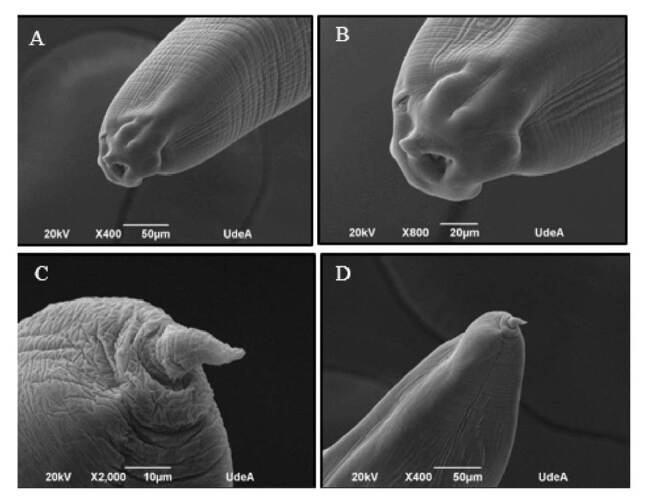
Microphotography of larvae of the genus *Anisakis* spp. (A) and (B) show the anterior portion of *Anisakis* spp. larvae. Anatomical structures are observed: presence of papillae, excretory pore, tooth, and ventral lip. (C) and (D) show the posterior portion of the larvae, with the presence of a mucron structure.

Identification was done under electron microscopy visualization, determining that the found larvae belonged to the genus *Anisakis* spp. (figure 1).

### Identification of Anisakidae larvae species

**Figure 2:**
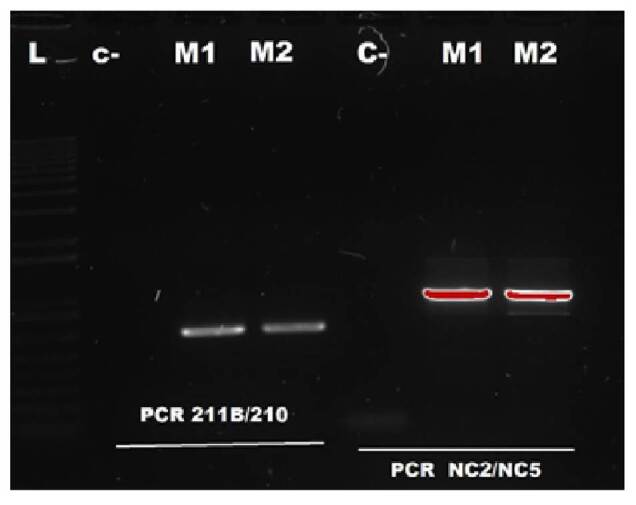
PCR results for the detection of *Anisakis* spp. Seven lanes are observed were the L-lane corresponds to the molecular weight marker or ladder; the second lane is the negative control of the PCR reaction -water; the third and fourth lanes are the samples M1 and M2 for the 211B/210 primers; and the fifth lane is the negative control for NC2/NC5 PCR amplification products.

**Figure 3:**
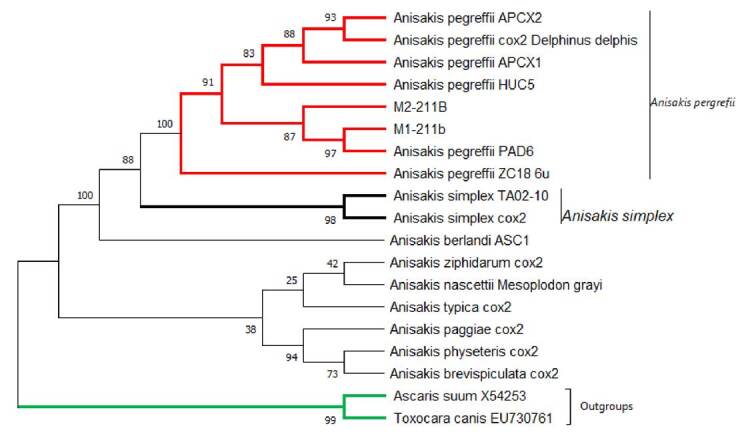
Phylogenetic tree of maximum likelihood comparison of *Anisakis* spp., based on the ITS1-5.8S-ITS2 region of rDNA.

Further molecular analysis confirmed the genus of the larvae as *Anisakis* spp. (figure 2) and *A. pegreffii* as the species. This could be established with the phylogenetic reconstruction of the analyzed sequences by means of maximum likelihood analysis, and collated the sequences reported in GenBank (figure 3).

## Discussion

This study aimed to determine the presence of larvae of the Anisakidae family in frozen raw fish fillets intended for human consumption, sold in three markets in Medellín and its metropolitan area. To the authors’ knowledge, this is the first study conducted in this area using ultraviolet light as larval identification method.

Worldwide, frequencies of up to 100% of fish analyzed have been reported. Similar data have been reported in fresh fish in Buenaventura and the Colombian Pacific coast ^21^^-^^23^, representing an important criterion for identification and quantification of these parasites in frozen raw fish. The reported frequency of Anisakidae larvae herein seems to be relatively low (1.04%), compared to studies. This low frequency found in the present study may be due to several conditions; one of which is the analyzed type of sample (fillet).

Considering different studies, it has been established that most Anisakidae worms are found in viscera from where they migrate to muscles, depending on the time it takes for evisceration ^24^^,^^25^. Reported ratios also differ in several studies. However, a 2:1 ratio could be found, decreasing the probability of finding larvae in muscle ^26^. In addition to this, it has been reported that *A. pegreffii* larvae have a lower penetration speed into the muscle, which could explain a lower presentation of worms at muscle level ^27^. Another reason for this frequency is the type of fish analyzed. Although the most consumed species in Medellin and its metropolitan area were analyzed, there are other reported types of fish with a higher presence of these parasites, that were not selected in the present study.

Such studies have been also carried out by a convenience sampling, which does not allow direct comparison of the data since the identification method differs greatly with respect to that used in the present study ^12^^,^^13^. Furthermore, it is known that the probability of finding larvae in frozen raw fish fillets is much lower than the probability of finding larvae in viscera ^28^, which would explain the low frequency found herein. However, this is the first study with a proportional-based design carried out on frozen fish fillets sold in different markets in Medellin and its metropolitan area. Therefore, our results demonstrate the existence of Anisakidae worms in frozen fish that are available for sale, which allows the assumption of a potential risk of parasitic transmission to humans.

No statistical association was found between the presence of *Anisakis* spp. and the explored variables (e.g., place of origin, market, type of fish), probably due to the relatively limited data. It is likely that a similar study using a larger sample size could show potential statistical associations. Nevertheless, the whole study reveals the significant risk of transmission to humans due to infected fish consumption, regardless of statistical association to a specific variable.

As stated before, the pressing technique followed by the ultraviolet light examination was used herein. This method has a high probability of detecting the presence of larvae in frozen fillets (sensitivity and specificity of 99%) ^29^^,^^30^, contributing to an efficient and economical way to accurately identify nematodes, such as those of the Anisakidae family. Similar studies have used this same method, detecting four *Hysterothylacium* spp. larvae in the analyzed samples, determining that ice crystals related to the freezing conservation method, contribute to the release of lipofuscin, an enzyme that generates a reaction to ultraviolet light ^30^^,^^31^. This is an accurate technique to identify Anisakidae nematodes when it is necessary to evaluate large batches of frozen fish prior to be commercialized for human consumption ^32^. This method is one of the most effective ones from an economic point of view, compared to others such as the enzyme digestion test, which has good sensitivity and specificity, but its high costs make this technique unfeasible as a frequent diagnostic alternative in the fishing industry ^29^^,^^30^.

It was possible to identify the presence of Anisakidae larvae, demonstrating the circulation of these nematodes in Medellin and its metropolitan area, which turns out to be relevant, since until now, there were no previous studies that reported the status of this parasitosis in the department of Antioquia. Through taxonomic analysis, it was possible to establish that the identified larvae present in the raw fish fillets belonged to the genus *Anisakis* spp., which are the most important species regarding parasitic human infections, due to the allergic and digestive symptoms they might trigger ^33^. Hence, the species described in the present study (*A. pegreffi*) reflects a potential risk for raw fish consumers, who could be infected with live larvae.

These findings are similar to previous reports ^12^^,^^13^ which state that the genus *Anisakis* spp. on the Pacific and Atlantic coasts of Colombia, as well as other genera, are of public health concern. From the epidemiological point of view, this is representative since the distribution of different fish species intended for human consumption comes from places where the finding is evident, which could represent potentially risky species for public health if they consumed raw or undercooked ^34^.

Since *A. pegreffi* has been detected in fish from the Colombian and Ecuadorian Pacific coasts ^14^, it could be established that fish that come from the Colombian Pacific could carry this species of *Anisakis* in products that are intended for human consumption. Similarly, although this species does not produce allergic reactions as *A. simplex*, it can cause severe gastroenteric symptoms in carriers, risking their lives ^6^^-^^8^. Most of the fillets that were analyzed in this study came from the port of Buenaventura, where the nematode has been reported at very high frequencies in different fish species intended for human consumption ^14^. In addition, fishing methods in Colombia are mainly artisanal ^35^, which increases the possibility of selling fish infected with these worms such as *Anisakis* spp., since they are sold without sanitary control and visual inspection from the health and food authorities.

According to the analysis of the study variables, the presence of *A. pegreffii* larvae in sawfish fillets come from the port of Buenaventura, which allows it to be classified as a potentially risky species when consumed raw in different culinary preparations such as *ceviche*^36^. *Anisakis pegreffii* and *A. simplex* are considered to share similar ways of causing disease in humans, a relevant situation to public health. Due to the gastrointestinal damage caused by the infection of the larvae, and the subsequent sequelae it leaves, it has been reported that these species of nematodes may be a risk factor for developing gastric cancer due to mucosal damage, and at the same time, trigger an immune reaction when in contact with the larvae antigens, though this last effect is still under study ^34^^,^^37^.

This report agrees with what was found in Mexico, where the Pacific sawfish was found to be one of the species that is most likely to be infected with *Anisakis* spp. ^38^. It should be noted that the capture method, selling, and habitat of this type of species in countries such as Mexico are like the processes carried out in Colombia, and that the presence of larvae may be subject to the time it takes to eviscerate, from capture to landing.

The results show a relatively low frequency, which cannot be considered epidemiologically significant. However, it is a starting point for further research on this nematode found in frozen fish. Additionally, only choosing four species of fish used for raw fish culinary preparations could have decreased the probability of finding a higher number of larvae, which, for future research, should be necessary to include risk groups and increase the number of fish species to be analyzed in order to better understand the behavior of the disease.

The present study is the first to report a frequency of 1.04% Anisakidae larvae compatible with the genus *Anisakis* and the species *pegreffii* in frozen raw fish fillets ready for human consumption in the department of Antioquia (Colombia). Our findings are of public health importance, revealing an important research approach in the field, oriented to expose the potential zoonotic risk and the impact that these parasitosis may have on the fishing industry and on human health. In addition, this type of study serves as an information mechanism for consumers, since they could prevent contamination from raw fish products using appropriate freezing, preservation, and cooking measures.
